# ADAPTING DIALECTICAL BEHAVIOR THERAPY TO ADDRESS CAREGIVER HEALTH IN HIGH-CONFLICT CAREGIVING SITUATIONS

**DOI:** 10.1093/geroni/igae098.1239

**Published:** 2024-12-31

**Authors:** Rachel VanPutten, Claudia Drossel, Thomas Waltz

**Affiliations:** VA Palo Alto Healthcare System, Palo Alto, California, United States; Eastern Michigan University, Ypsilanti, Michigan, United States; Eastern Michigan University, Ypsilanti, Michigan, United States

## Abstract

Family caregivers of older adults with neurocognitive disorders (NCDs) are invisible patients in the U.S. healthcare system. This is especially concerning because poor caregiver mental or physical health predicts coercive care practices (e.g., yelling or threatening the older adult). When older adults with NCD come to providers’ attention for emotional and behavioral changes, systematic strategies for assessing and intervening on caregiver health and potential coercive caregiving patterns are lacking. The present study consisted of a retrospective chart review of clinical records from family caregivers who participated in dialectical behavior therapy (DBT) skills training groups adapted for high-conflict situations involving older adults with NCDs (Drossel, Fisher, & Mercer, 2011) in a doctoral-level psychology training clinic in the Midwest. Data from 32 family caregivers enrolled across eight 8-week DBT groups showed that caregivers whose medical records indicated significant health problems tended to describe themselves as healthy when completing the RAND 36-Item Health Survey (SF-36; Hayes et al., 1993). Attrition from the groups was mainly due to acute medical crises, and caregivers required significant individual support to address their medical concerns, prompting modifications to the DBT mindfulness and interpersonal effectiveness modules (e.g., developing DEAR MAN scripts to delegate care tasks to third-party providers). These findings suggest the need for routine, systematic, and direct assessments of caregiver health and skills-based approaches targeting health promotion and disease prevention to address coercive caregiving practices.

